# Appropriate dose of intravitreal ranibizumab for ROP: a retrospective study

**DOI:** 10.1186/s12886-022-02489-6

**Published:** 2022-06-21

**Authors:** Yingying Chen, Shaoli Wang, Siying Chen, Xingyue Chen, Lizhen Han, Qionglei Zhong, Kaiyan Zhang

**Affiliations:** Hainan General Hospital , Hainan Affiliated Hospital Of Hainan Medical University, Xiuhua road, Hainan General Hospital, Hainan, China

**Keywords:** Retinopathy of prematurity, Ranibizumab, Dose, Vascular endothelial growth factor, Recurrence rate

## Abstract

**Objective:**

To compare the recurrence rate of retinopathy of prematurity (ROP) after treatment with 0.3 mg vs. 0.25 mg ranibizumab.

**Subjects:**

All patients with ROP who underwent intravitreal injection of ranibizumab in Hainan General Hospital between January 2014 and May 2020 were included in this retrospective study.

**Methods:**

Eighty-two cases (146 eyes) who received intravitreal injection of 0.25 mg ranibizumab were included in the conventional-dose group, and 59 cases (108 eyes) who received intravitreal injection of 0.3 mg ranibizumab were included in the high-dose group. The two groups were further divided into the 25-28-week, 29-31-week, 32-34-week, and 35-36-week GA subgroups. The differences between the conventional-dose group and the high-dose group in gestational age (GA), birth weight (BW), age at initial injection (weeks), incidence of systemic diseases, the recurrence rate of ROP, and age at retinal vascularization completed (weeks) were analyzed.

**Results:**

GA, BW, age at initial injection, and the incidence of systemic diseases were not significantly different between the conventional-dose group and the high-dose group (*p* > 0.05). The recurrence rates of ROP were significantly lower in the 25-28-week, 29-31-week, and 32-34-week subgroups of the high-dose group than in the same subgroups of the conventional-dose group (*p* < 0.05). Within the conventional-dose group, the recurrence rate of ROP was significantly lower in the 32-34-week and 35-36-week subgroups than in the 25-28-week and 29-31-week subgroups (*p* < 0.05). Within the high-dose group, the recurrence rate of ROP was not significantly different between the four subgroups (*p* > 0.05). Retinal vascularization was completed at a later age in the 32-34-week subgroup of the high-dose group than in the 32-34-week subgroup of the conventional-dose group (*p* < 0.05) but was not significantly different between the two groups at any other GA range (*p* > 0.05). No severe ocular or systemic complications occurred in any patient.

**Conclusion:**

Treatment with 0.3 mg ranibizumab can reduce the recurrence rate of ROP without prolonging retinal vascularization or causing serious systemic complications. Therefore, this dose may be an appropriate therapeutic dose for ROP.

## Introduction

Retinopathy of prematurity (ROP) is an eye disease caused by vascular proliferation in the immature retina of premature infants. First described by Terry [1] in 1942, as a blinding eye disease that seriously threatens children’s vision. The prevalence of ROP varies between countries. Gerd [2] et al. found that the incidence of ROP was 31.9% in Sweden between 2008 and 2015. Sarah [3] et al. showed that the prevalence of ROP that required treatment in Northern Ireland increased from 1.05% in 2000 to 5.78% in 2011. Siswanto [4] et al. conducted a study in Indonesia and found that the prevalence of ROP in infants with gestational age (GA) < 32 weeks was 18-30% at different stages of the study. The pathological process of ROP comprises two stages: vascular growth arrest and secondary vascular proliferation. The first stage mainly occurs from birth to the corrected age of 30 weeks. In this stage, the preterm infant leaves the relatively hypoxic environment of the uterus for the relatively high-oxygen environment outside the maternal body. The loss of maternal–fetal interaction and the inhibition of vascular endothelial growth factor (VEGF) and other proangiogenic factors in the high-oxygen environment hinder retinal vascularization. The second stage occurs at the corrected age of 31-42 weeks. As the metabolic demand of retinal development continuously increases, the poorly growing blood vessels cannot meet the oxygen demand of the tissues, which stimulates pathologically excessive production of VEGF and other proangiogenic factors, resulting in abnormal retinal angiogenesis and leading to the occurrence and progression of ROP [5, 6].

According to the classification criteria developed by the Committee for the Classification of Retinopathy of Prematurity in 1984 [7], ROP is divided into 3 zones, and is classified into 5 stages based on the disease course. Plus disease refers to dilatation and tortuosity of retinal vessels in at least 2 quadrants at the posterior pole. The “+” is used to represent the presence of plus disease. Pre-threshold ROP is classified into types 1 and 2 [8]. Type 1 ROP includes zone I ROP of any stage with plus disease(+),zone I ROP of stage 3 without plus disease, and zone II ROP of stage 2 and 3 with plus disease(+). Type 2 ROP include zone I ROP of stage 1 and 2, zone II ROP of stage 3 without plus disease. According to ETROP (Early treatment for Retinopathy of Prematurity Cooperative Group), all cases of type 1 ROP should be treated [8, 9].

Laser treatment is the gold standard for the treatment of type 1 ROP [8], but it may cause peripheral visual field defects, refractive errors, and peripheral traction retinal detachment [10]. In recent years, intravitreal injection of VEGF inhibitors has attracted great attention as a new treatment method for ROP. VEGF inhibitors have been proven to significantly reduce the neovascular response and the incidence of refractive errors without damaging the peripheral retina, making it more advantageous than traditional treatment methods such as retinal cryotherapy or laser treatment [11–17]. The BEAT-ROP [18] trial showed that bevacizumab, a VEGF inhibitor, is effective for zone I ROP In 2011. At the same year, ranibizumab was approved by the Chinese Food and Drug Administration (CFDA) for the treatment of age-related macular degeneration (AMD) as a VEGF inhibitor. Luis [19] et al. (2011) and Castellanos [20] et al. (2013) started to use ranibizumab in the treatment of ROP and achieved satisfactory therapeutic effects. Xu [21] et al. started to apply ranibizumab in treating ROP in China in 2012. In January 2014, we started to perform intravitreal injection of ranibizumab in ROP patients in the Department of Neonatology of our hospital. At that time, there was no consensus on the optimal dose of VEGF inhibitors for the treatment of ROP [22–24], but most infants were given half the therapeutic dose for adults. Following this practice, we used a single injection of ranibizumab at a dose of 0.25 mg/0.025 ml per eye from January 2014 to August 2017 to treat ROP. However, Erol [25] et al. and Wong [26] et al. used the same dose of ranibizumab to treat ROP and found high recurrence rates of ROP after treatment. Whereas In 2012, Mota [27] et al. gave 0.3 mg/0.03 ml ranibizumab for the treatment of aggressive posterior retinopathy of prematurity (APROP) and reported no ocular or systemic adverse reactions. Therefore, since September 2017, we switched to a single injection of ranibizumab at a dose of 0.3 mg/0.03 ml per eye.

## Research method

We retrospectively studied the ROP patients who underwent intravitreal injection of ranibizumab between January 2014 and May 2020 in the Department of Neonatology, Hainan General Hospital and the patients were followed up till the corrected age of 50 weeks or the retinal vessels reaching the temporal ora serrata. All 82 cases who received intravitreal injection of ranibizumab between January 2014 and August 2017 were included in the conventional-dose group. All 59 cases who received intravitreal injection of ranibizumab between September 2017 and May 2020 were included in the high-dose group.

According to the standard procedure of intravitreal injection [28], antibiotic eye drops (Tobramycin eye drops) should be used for 3 days before intravitreal injection. Since the patients with type 1 ROP in this study underwent the operation within 24 hours of diagnosis, they received eye drops 6-8 times the day before operation in accordance with the “Expert advice on the standardization of perioperative infection prevention measures for cataract [29]. Intravitreal injection was performed in a sterile operating room. Before injection, proparacaine hydrochloride eye drops were used for surface anesthesia of each eye to be injected, and a 10% povidone-iodine solution was used to disinfect the skin around the eye. Then, push the drug to the dose scale of the required injection in advance. Open the eye with a blepharostat, instill 5% povidone-iodine eye drops into the conjunctival sac of the eye. After 90 seconds, the conjunctival sac was rinsed with normal saline. Next, ranibizumab was injected at 1.5 mm posterior corneal limbus. After the injection, a sterile cotton swab was used to press the top of the injection site for 1 minute. If the other eye was to be treated, all surgical instruments were replaced. After the operation, the operated eye was given tobramycin and dexamethasone ophthalmic ointment and covered with gauze, which was removed the day after surgery, and the operated eye was instilled with antibiotic eye drops four times a day for 3 days [30]. On the first postoperative day, the fundus was examined under pupillary dilation using an indirect ophthalmoscope to observe whether there was central retinal artery occlusion, retinal or vitreous hemorrhage, retinal tear or detachment, or lens injury. The fundus was examined weekly in the first month after operation, once every 2 weeks in the second month, and once a month from the third month until the complete vascularization of the retina [31] or the termination of retinal vessels in zone II or III without pathological vascular hyperplasia or retinal proliferation. The operation and follow-up examinations were performed by the same ophthalmologist.

The two groups were further divided into the 25-28-week, 29-31-week, 32-34-week, and 35-36-week GA subgroups. The differences between the conventional-dose group and the high-dose group in gestational age (GA), birth weight (BW), age at initial injection (weeks), incidence of systemic diseases, the recurrence rate of ROP, and age at retinal vascularization completed (weeks) were analyzed. ROP related systemic diseases include respiratory distress syndrome (RDS), intraventricular hemorrhage (IH), septicemia, anemia, Maternal diabetes mellitus, etc. [32–36]. Recurrence has been defined as reappearance of plus disease, neovascularization, extraretinal fibrovascular proliferation, new ridge after prior initial regression, or progression of disease despite prior treatment [37]. Retinal vascularization completed was defined as the retinal vessels reaching the temporal ora serrata [38]. In this study, the retinal vessels of 3 patients terminated in zone II or III, so they were excluded from the analysis of retinal vascularization completed. All data were analyzed with SPSS and are expressed as mean ± standard deviation. GA, BW, age at initial injection, and age at retinal vascularization completed were compared between the two groups with the independent-samples t-test. Incidence of systemic diseases were compared between the two groups by the chi-squared test. The recurrence rate of ROP was compared pairwise between the four GA subgroups of the conventional-dose group and the high-dose group using the chi-squared and Fisher’s exact test. *p* < 0.05 was considered statistically significant.

## Results

From January 2014 to May 2020, a total of 3822 premature infants (7644 eyes) in the Department of Neonatology of our hospital were screened and 141 patients (254 eyes) received intravitreal injection of ranibizumab, including 133 cases of type 1 ROP and 8 cases of APROP. The 82 cases (146 eyes) who received intravitreal injection of ranibizumab between January 2014 and August 2017 were included in the conventional-dose group, including 17 patients (30 eyes) in the 25-28-week subgroup, 21 patients (36 eyes) in the 29-31-week subgroup, 31 patients (57 eyes) in the 32-34-week subgroup, and 13 patients (23 eyes) in the 35-36-week subgroup. The 59 cases (108 eyes) who received intravitreal injection of ranibizumab between September 2017 and May 2020 were included in the high-dose group, including 14 patients (26 eyes) in the 25-28-week subgroup, 17 patients (31 eyes) in the 29-31-week subgroup, 21 patients (40 eyes) in the 32-34-week subgroup, and 7 patients (11 eyes) in the 35-36-week subgroup. The numbers of ROP patients and eyes in each GA subgroup of the conventional-dose and high-dose groups are listed in Table 1.

**Table 1:** Numbers of ROP patients and eyes in each GA subgroup of two groups

GA (wk)	conventional-dose group	high-dose group
25 ~ 28	17/30	14/26
29 ~ 31	21/36	17/31
32 ~ 34	31/57	21/40
35 ~ 36	13/23	7/11

The two groups were not significantly different in GA, BW, age at initial injection (*p* > 0.05) (Table 2), or the incidence of systemic diseases (*p* > 0.05) (Table 3).

**Table 2:** Demographic data for two groups

	conventional-dose group	high-dose group	t-value	*p*-value
GA (wk)	31.93 ± 3.11	31.14 ± 3.17	1.486	0.139
BW(g)	1158.29 ± 351.06	1072.54 ± 328.37	1.470	0.144
Age at initial injection (wk)	34.64 ± 1.61	34.18 ± 1.63	1.692	0.093

**Table 3:** Incidence of systemic diseases for two groups

	Respiratory distress syndrome	intraventricular hemorrhage	septicemia	anemia	heart disease	Maternal diabetes mellitus	Maternal preeclampsia
conventional-dose group(82cases)	74 (90.24)	29 (35.37)	24 (29.27)	39 (47.56)	29 (35.37)	18 (21.95)	28 (34.15)
high-dose group(59cases)	50 (84.75)	28 (47.46)	17 (28.81)	34 (57.63)	25 (42.37)	16 (27.12)	22 (37.29)
X2	0.978	2.083	0.003	1.392	0.713	0.501	0.148
*p*-value	0.323	0.149	0.953	0.238	0.398	0.479	0.700

In the 25-28-week subgroups, ROP relapsed in 9 (30.0%) eyes in the conventional-dose group vs. 2 (7.69%) eyes in the high-dose group (*p* < 0.05). In the 29-31-week subgroups, ROP relapsed in 10 (27.78%) eyes in the conventional-dose group vs. 2 (6.45%) eyes in the high-dose group (*p* < 0.05). In the 32-34-week subgroups, ROP relapsed in 6 (10.53%) eyes in the conventional-dose group vs. 0 (0%) eyes in the high-dose group (*p* < 0.05). In the 35-36-week subgroups, ROP relapsed in 1 (4.35%) eye in the conventional-dose group vs. 0 (0%) eyes in the high-dose group (*p* > 0.05) (Table 4).

**Table 4:** The recurrence rates of ROP in each GA subgroup of two groups

GA (wk)	conventional-dose group	high-dose group	X2	*p*-value
25 ~ 28	9 (30.00)	2 (7.69)	4.391	0.047
29 ~ 31	10 (27.78)	2 (6.45)	5.153	0.028
32 ~ 34	6 (10.53)	0 (0)	4.488	0.041
35 ~ 36	1 (4.35)	0 (0)	0.599	1.000

The age at retinal vascularization completed was later in the 32-34-week subgroup of the high-dose group than in the 32-34-week subgroup of the conventional-dose group (*p* < 0.05) but was not significantly different between the two groups at any other GA range (*p* > 0.05) (Table 5).

**Table 5:** The age at retinal vascularization completed in each GA subgroup of two groups

GA (wk)	conventional-dose group	high-dose group	t-value	*p*-value
25 ~ 28	49.86 ± 0.89	49.36 ± 0.77	1.639	0.112
29 ~ 31	51.24 ± 0.58	50.98 ± 0.64	1.350	0.185
32 ~ 34	48.38 ± 0.81	48.85 ± 0.83	2.029	0.048
35 ~ 36	49.01 ± 0.79	48.50 ± 1.07	1.215	0.240

Within the conventional-dose group, the recurrence rate of ROP was significantly lower in the 32-34-week and 35-36-week subgroups than in the 25-28-week and 29-31-week subgroups (*p* < 0.05). Within the high-dose group, the recurrence rate of ROP was not significantly different between the four subgroups (*p* > 0.05).

Among the 146 eyes in the conventional-dose group, ROP relapsed in 26 eyes, the patients with recurrent ROP received the second intravitreal injection and the lesions subsided, no patients need the third injection or laser treatment. Among the 108 eyes in the high-dose group, ROP subsided in 3 eyes after the second intravitreal injection and in 1 eye after the third intravitreal injection, but no patients need laser treatment.

Bui [39] et al. found that the acute increase in intraocular pressure (IOP) exceeded 15 mmHg within 105 minutes after intravitreal injection, which could lead to permanent dysfunction in the animal model, as evidenced by electroretinograms (ERG). The IOP generally peaked within 5 minutes after intravitreal injection [40]. We observed that nearly all patients in the high-dose group developed corneal edema in the eye injected with 0.3 mg/0.03 ml ranibizumab immediately after injection, which manifested as a slight whitening of the cornea, but the transparency of the cornea was restored within 5 seconds. Therefore, the IOP was not continuously rising, so no IOP-lowering treatment, such as paracentesis of anterior chamber, was performed. None of the patients had operation-related complications, such as central retinal artery occlusion, retinal or vitreous hemorrhage, retinal tear or detachment, or iatrogenic cataract, or postoperative systemic complications.

## Discussion

VEGF inhibitors have high efficacy against ROP. In 2019, the European Commission approved the application of ranibizumab, a VEGF inhibitor for treating ROP. Ranibizumab is a specially designed recombinant humanized antibody, which can bind to and inhibit all biologically active VEGF subtypes [41]. Avery [42] et al., Zehetner [43] et al., and Carneiro [44] et al. found that after intravitreal injection of ranibizumab in adult patients with AMD or diabetic retinopathy (DR), serum VEGF did not decrease. Hoerster [45] et al. found that the serum VEGF in patients with ROP decreased 2-3 weeks after intravitreal injection of ranibizumab and returned to normal at 4 weeks. Ranibizumab has a higher binding affinity to VEGF than bevacizumab. Therefore, theoretically, ranibizumab is superior to bevacizumab for preterm infants in terms of therapeutic effect and side effects after systemic absorption [26]. Castellanos [20] et al. used ranibizumab for ROP treatment and followed up the patients for 3 years, and their findings supported the efficacy and safety of ranibizumab.

There is no consensus on the optimal doses of VEGF inhibitors for ROP. In the BEAT-ROP trial, Mintz-Hittner [18] recommended the use of 0.625 mg of bevacizumab for children, which is half of the adult dose. Spandau [46] used 0.4 mg of bevacizumab. Han [47] believed that 0.25 mg was an effective dose for bevacizumab. Connor [48] et al. injected 0.16 mg of bevacizumab into one eye of a patient with ROP and 0.32 mg of bevacizumab to the other eye of the same patient then found that 0.16 mg was enough to effectively treat ROP.

Honda [49] and Zepeda [50] had confirmed that VEGF inhibitors cannot be used for stage 4 or 5 ROP because they can increase the contraction of the fibrovascular membrane and accelerate retinal detachment. Hainan General Hospital has been carrying out standard screening of ROP since 2010. In that time, all confirmed ROP cases in the Department of Neonatology were of stages 1-3, and no patient was diagnosed with stage 4 or 5 ROP. From January 2014 to August 2017, all intravitreal injections of ranibizumab in pediatric patients were at half of the adult dose, namely, 0.25 mg. However, during the follow-up, ROP relapsed in some patients at 2 to 3 weeks after the operation. The recurrence rate of ROP after treatment with 0.25 mg ranibizumab was 40% in the study of Erol [25] et al. and 83% in the study of Wong [26]. In China, patients need to pay all of the cost of ROP treatment with VEGF inhibitors, so the high cost of VEGF inhibitors puts a great burden on the family of patients with ROP recurrence. With the goal of ensuring safety, we tried to reduce the recurrence rate of ROP by increasing the dose of ranibizumab. We found that Mota [27] et al. reported no systemic complications in patients who received 0.3 mg/0.03 ml ranibizumab for the treatment of APROP. Therefore, after obtaining the approval of the ethic committee of Hainan General Hospital and the consent from the patients’ family members, the injection dose of ranibizumab was increased to 0.3 mg in September 2017, and that dose has been used in our hospital since then. According to the study of Meng [30] at Peking University People’s Hospital, patients with recurrent ROP with plus disease(+)could receive repeated intravitreal injections of VEGF inhibitors, our recurrent ROP patients with plus disease(+)included in the present study all received intravitreal injection of 0.3 mg/0.03 ml ranibizumab to avoid laser-induced ocular complications after repeated full communication with the family members of the patients.

The results of this retrospective analysis showed that GA, BW, and age at initial injection were lower, but not significantly, in the high-dose group than in the conventional-dose group, which we speculate was related to the increasing mean age of puerpera and the decreasing GA of preterm infants since China started to abolish the one-child policy in 2016. ROP related systemic diseases include RDS, IH, septicemia, anemia, etc. were not significantly different between the high-dose group and the conventional-dose group. In the high-dose group, the recurrence rate of ROP was not significantly different between the four subgroups. Except in the 35-36-week subgroups, the recurrence rates of ROP in the GA subgroups of the high-dose group was significantly lower than those in the same subgroups of the conventional-dose group, perhaps because the reduction in VEGF level by the high injection dose promoted the disappearance of neovascularization. ROP did not recur in the 32-34-week or 35-36-week subgroup of the high-dose group. The recurrence rates of ROP in the 32-34-week and 35-36-week subgroups of the conventional-dose group were significantly lower than the rates in the 25-28-week and 29-31-week subgroups of the same group. Erol [25] et al. compared the efficacy of ranibizumab and bevacizumab in the treatment of ROP and thought the higher recurrence rate of ROP after ranibizumab injection than after bevacizumab injection was the result of the different pharmacokinetic behaviors caused by incomplete organ development in preterm infants. Tolentino [51] and Avery [52] have pointed out that the half-life of ranibizumab is short (the systemic half-life of ranibizumab is approximately 2 hours in adults). Therefore, we infer that in the conventional-dose group, the higher recurrence rate of ROP in patients with a lower GA (the 25-28-week and 29-31-week subgroups) than in patients with a higher GA (the 32-34-week and 35-36-week subgroups) was likely because of the reincrease in VEGF concentration due to incomplete vascularization of the retina after ranibizumab metabolism in patients with a lower GA. Theoretically, the retinal vessels should reach the temporal ora serrata at 40 weeks of GA [53]. VEGF inhibitors can prolong retinal vascularization, and the peripheral retinal avascular area will remain for several years in some patients after the use of VEGF inhibitors [12, 54]. However, according to our follow-up results, except for 3 patients with retinal vessels ending in zone II or III, all our patients had peripheral retinal vessels reaching the temporal ora serrata, and except that the age at retinal vascularization completed in the 32-34-week subgroup of the high-dose group was significantly later than that of the 32-34-week subgroup of the conventional-dose group, there was no significant difference in age at retinal vascularization completed between any subgroup of the high-dose group and the same subgroup of the conventional-dose group, indicating that high-dose VEGF inhibitors did not prolong retinal vascularization.

VEGF plays an important role in the development of many organs. VEGF is expressed in the lungs, kidneys, and brains of neonates [55]. Most notably, VEGF is involved in alveolar development and lung maturation [56]. According to the analysis of the systemic conditions of children with ROP in this study, no patients died and no new-onset systemic complications or exacerbations of existing systemic diseases like lung dysplasia occurred. Although some scholars have shown that intravitreal injection of VEGF inhibitors may reduce the serum VEGF in patients with ROP, the BEAT-ROP trial found that VEGF inhibitors do not increase the mortality of children [18]. Seyhan [57] et al. did not observe any local or systemic side effects in children treated with bevacizumab, which is consistent with the results of our study.

## Conclusion

The purpose of this article was to investigate whether the recurrence rate of ROP can be reduced by increasing the dose of ranibizumab. The results showed that 0.3 mg ranibizumab can reduce the recurrence rate of ROP without prolonging retinal vascularization or causing systemic complications. Therefore, 0.3 mg ranibizumab could be considered for the treatment of ROP.

## Data Availability

The datasets generated and/or analyzed during the current study are not publicly available (In order to protect the author’s article property rights, it can be provided after publication) but are available from the corresponding author on reasonable request.
